# Comparison effect of physiotherapy with surgery on sexual function in patients with pelvic floor disorder: A randomized clinical trial

**Published:** 2014-01

**Authors:** Tahereh Eftekhar, Maryam Sohrabi, Fedyeh Haghollahi, Mamak Shariat, Elahe Miri

**Affiliations:** 1 *Institute for Family Health, Vali-e-Asr Reproductive Health Research Center, Vali-e-Asr Hospital, Tehran University of Medical Sciences, Tehran, Iran.*; 2 *Gynecology Ward, Vali-e-Asr Hospital, Tehran University of Medical Sciences, Tehran, Iran.*; 3 *Institute for Family Health, Maternal-Fetal and Neonatal Research Center, Tehran University of Medical Sciences, Tehran, Iran.*

**Keywords:** *Pelvic floor*, *FSFI*, *Sexual dysfunction*, *Physiotherapy*, *Physical Therapy*, *Pelvic surgery*

## Abstract

**Background:** Female sexual dysfunction is a common problem among general population, especially in urogynecological patient, and can lead to a decrease in quality of life and affect martial relationship.

**Objective: **This study was compared the effect of surgical methods versus physiotherapy on sexual function in pelvic floor disorder.

**Materials and Methods:** This randomized controlled trial was performed in Urogynecology clinic since August 2007 to December 2009 on 90 patients aged from 25-55 years with previous delivery, positive history of sexual dysfunction with stage <3 of pelvic organ prolapsed and divided in two groups. Group A (n=45) received standard rectocele repair and prineorrhaphy, group B (n=45) received physiotherapy for eight weeks twice a week (electrical stimulation, Kegel exercises). The female sexual function index (FSFI) used to evaluate the sexual function in cases before and after intervention. Frequency of variable scores (libido, orgasm, dysparunia) included without disorder, frequently good, sometimes good, very much and extreme were compared between two groups.

**Results:** Libido and arousal were improved in both groups (p=0.007, p=0.001 respectively). Orgasm and dyspareunia were improved in group B (p=0.001). Dysparunia was more painful in group A. There was significant difference between two groups (improvement of orgasm and dysparunia in group B) (p=0.001).

**Conclusion:** It seems that physiotherapy is an appropriate method for treatment of sexual disorder in pelvic floor disorder.

Registration ID in IRCT: IRCT2013031112790N1.

## Introduction

Pelvic floor includes several layers from top to low; surrounding connective tissue from endopelvic fascia, pelvic diaphragm (levator ani and coccygeous muscles), perineal membrane (urogenital diaphragm), superficial layer (ischiocavernous, bulbocavernous and superficial transverse perineal muscles) (1). These structures would protect the visceral organs. Pelvic floor dysfunction (PFD), a disease with anatomical and/or functional abnormalities of pelvic organs due to weakened supporting tissues of the pelvic floor and dislocation of pelvic organs that may result in lower urinary tract and lower gastrointestinal tract disorders. Also the pelvic floor is a strong factor contributing for genital function and behavior in men and women (2). Female sexual dysfunction is highly prevalence (11%) and may affect the anterior, posterior, or apical compartment with a negative impact on sexual function (3).

A sexual problem, or sexual dysfunction, refers to a problem during any phase of the sexual response cycle that prevents the individuals or couple from experiencing satisfaction from the sexual activity and resulting from physical, social, and psychological factors. The sexual dysfunction stages include libido disorder (decreased sexual thoughts), arousal disorder (decreased ability to obtain or maintain sexual excitement), orgasm disorder and dyspareunia (4). Women with sexual distress are more likely to report sexual difficulty related to pelvic floor symptoms, including sexual avoidance due to vaginal prolapse or sexual activity restriction due to fear of urinary incontinence (UI) (5). Pelvic organ prolapse (POP) is associated with significant bother, distress and decreased quality of life (QoL) (6). Surgical methods, rectocele and prineorrhaphy include levator muscle plication, deep repair of puborectalis muscles, and perineal enforcement may result in constriction of middle vaginal region and dyspareunia (7). 

Surgical correction of POP and UI improves sexual function in approximately 70% of patients, although some studies show no change with the use of non-condition-specific questionnaires (8). So the physicians and researchers are seeking for alternative surgical methods for pelvic floor dysfunction. One of these alternative methods is pelvic floor physiotherapy that was firstly introduced by Arnold Kegel (9). Pelvic exercises result in increased pubococcygeous tonicity and improved orgasm power, and correction of UI and would result in hyper arousal and congestion and increase in genital sensation and more favorable orgasm during intercourse (10). In an RCT, the pelvic floor exercise resulted in improved quality of life and sexual function in women with UI (11). In a study in Turkey, the improvement of libido and orgasm during intercourse after pelvic floor exercise was reported (12).

Based on the few data available there is strong evidence for the efficacy of physical therapy for the treatment stress UI in women but further studies are needed to evaluate the optimal training protocol and length of treatment (13). Also the sexual dysfunction may result in psychological and sexual problems among women, and comparative study is not performed, between surgical and physiotherapy methods on sexual function in pelvic floor dysfunction treatments. Therefore this study was compared the effect of surgical methods versus physiotherapy on sexual function in pelvic floor disorder.

## Materials and methods

This study was performed as a randomized clinical trial among 90 women aged from 25-55 years, BMI ranged from 18-30 with previous delivery, positive history of sexual dysfunction, and with grade <3 of pelvic organ prolapsed based on pelvic organ prolapse quantification (POP-Q examination) in Urogynecology clinic of Vali-e-Asr Hospital of Tehran university of medical sciences from 2007 through 2009. Pelvic organ prolapse was defined the most distal portion of the prolapse is more than 1 cm above the level of the hymen (stage 1) and the most distal portion of the prolapse is 1 cm or less proximal or distal to the hymenal plane(stage 2) as measured on clinical exam (2).

**Figure 1:**
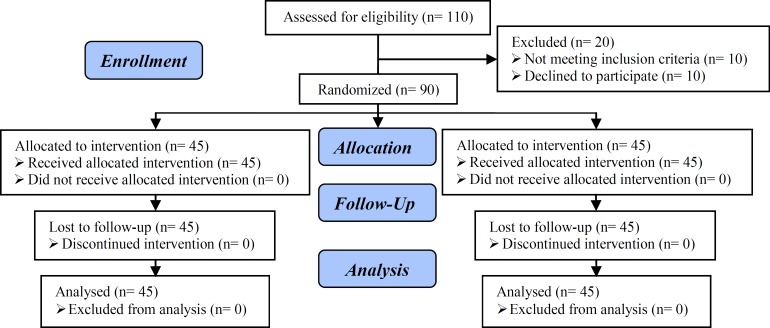
Trial Flow Chart

From 110 patients with PFD, exclusion of (n=10) and 90 patients were included. After obtaining written consent they were allocated by the clinic secretary to one of two groups by random sampling, using a random numbers table into two groups of 45 patients (Group A)received a surgical repair pelvic relaxation (standard rectocele repair and prineorrhaphy), and group B received physiotherapy, eight weeks twice a week (electrical stimulation, Kegel exercises) (Figure 1). The exclusion criteria were single status, history of systemic and psychological diseases, history of pelvic floor surgery, high grade prolapse (grade3, 4), and muscle relaxant and psychoactive drugs use. The patients had knowledge about the treatment assignment. Before the project commenced, the survey was approved by Ethics Committee of Tehran University of Medical Sciences. 

The physiotherapies included vaginal and anal biofeedback, infra red, reinforcement exercises, and relaxation including Kegel exercises. The electrical stimulation was used for enforcement and recognition of pelvic floor muscles and cooperation between them and then the Kegel exercises were trained to be performed in the home to maintain the lifelong efficacy of exercise and improve the quality of life. Kegel exercises were included slow and quick contractions for three times a day. Kegel exercise was taught to patients (by investigator); consisted of 6-8 sec of contractions with 6 sec rest in between, for 15 min, three times a day, for a total duration of 8 weeks. After the informed consent was fulfilled, then the general questionnaire including demographic and obstetric characteristics and the sexual function questionnaire were fulfilled (3). 

The female sexual function index (FSFI) was fulfilled before and eight weeks after the intervention. The patients in surgery group (group A) had no permission for intercourse for six weeks and then were permitted and sexual function index was fulfilled again. In group B, two months after the intervention, the female sexual function index was fulfilled again. In this questionnaire, the first two questions were about the libido (sexual desire) or interest is a feeling that includes wanting to have a sexual experience, feeling receptive to a partner's sexual initiation, and thinking or fantasizing about having sex. 

The second eight questions were about arousal that four were about lubrication (vaginal arousal) and four were about warmness and palpitation sensation in genitalia (arousal sensation), and also there were three questions about orgasm, three about satisfaction sense, three about dysparunia. Each question had five responses including without disorder, most of good, sometimes good, very much, and extreme and or never, not at all, sometimes, often, and always with a score range of zero to five. The individual domain scores of the FSFI is described in the table I. Variable scores (libido, orgasm, dysparunia) was compared between two groups. 

P<0.05 were considered significant. The highest and lowest scores for libido were 10 and 2 and for arousal were 40 and 0. Also the highest and lowest scores for orgasm were 15 and 0 and for dysparunia were 15 and 0 respectively. The scores for libido ranged from 6-9 were frequently good, 3-5 were sometimes good and 2 were severing disorder. The scores for arousal ranged from 25-39 were frequently good and 5-24 were sometimes good. The scores for orgasm ranged from 0-15 were without disorder, 9-11 were frequently good, 3-8 were sometimes good, and less than 3 were severe disorder. The scores for dyspareunia ranged from 12-15 were without disorder, 9-12 were frequently good, 3-9 were sometimes good, and less than 3 were considered as severe disorder. 

**Statistical analysis**

The final statistical analysis was performed using SPSS version 15.0 software. The ratio frequency was used for categorical variables and the mean and standard deviation was used for numerical variables. The ratios were compared by Chi-Squared test, Fisher Exact tests and Wilcoxon Rank test and the means were compared by Student's *t*-test. Multinomial Regression was used to assess the effect of variables on sexual function factors. p<0.05 was considered significant.

## Results

In this study, age, weight, BMI was similar between two groups. However, primary school education and the number of parity were different in two groups (Table II). Despite the fact that education level in physiotherapy is more than surgery group, and number of housewife women is lower, but multinomial regression test showed that these two factors are not affected responding patients in sexual function, respectively (p=0.078, p=0.27). Although the number of births in the two groups is statistically significant. But delivery type (cesarean, normal vaginal delivery) had no statistically significant difference between two groups. The type of previous delivery was normal vaginal in 34 patients (75.6%) in surgery group and 32 subjects (71.1%) in control group (p=0.315). 

It should be noted that urge, gas and fecal incontinencyhad no significant difference before intervention in two groups (p=0.20, p=0.30, p=0.28 respectively). Sexual variables (arousal, libido, orgasm, dysparunia) were compared in two groups (Table III). After the intervention in physiotherapy group sever orgasm disorder was reduced from 7 (15%) to 1 (2%) and 33 patients (74%) were improved to normal condition included (without disorder, Most of good). In the surgical group, sever orgasm disorder in 2 patients (5%) was changed to 9 (15%) after intervention. Analytical t-test showed that Improvement of severe orgasm disorder had significant difference in two groups after intervention (p=0.001). 

In the other words, physical therapy improved orgasm compared to surgical group in physiotherapy group after the intervention. Sever dyspareunia disorder was reduced from 13 (29%) to 6 (13%) and in 27 patients (60%) were improved to normal condition included (without disorder, Most of good). In the surgical group, sever dyspareunia disorder in 32 patients (71%) is changed to 37 (82%) after intervention. The data showed that there was significant increase in dyspareunia (p=0.001). Libido and arousal were improved in both groups (p=0.007, p=0.001 respectively). Orgasm was improved in physiotherapy group (p=0.001). There was significant difference between two groups (improvement of orgasm and dyspareunia) (p=0.001). Totally, physiotherapy improved significantly libido, arousal, orgasm, and dyspareunia (p=0.001) (Table III).

**Table I:** Domain Scoring

**Domain**	**Questions**	**Score range**	**Min**	**Max**
Desire 1	1, 2	1-5	2	15
Arousal^2^	3, 4, 5, 6, 7, 8, 9, 10	0-5	0	40
Orgasm^3^	11, 12, 13	0-5	0	15
Satisfaction	14, 15, 16	1-5	1	15
Dysparunia^4^	17, 18, 19	0-5	0	15

1. desire for sexual activity

2. desire for sexual activity with sexual stimulations

3. to reach orgasm after adequate sexual arousal and stimulation

4. pain in the pelvis or vagina during any stage of normal sexual stage

**Table II:** Baseline characteristics in group A and B

** Groups**			**Surgery (A group)**	**Physiotherapy (B group)**	**p-value**
**Variable**					
*Age (year)			37.7 ± 5.8	35.4 ± 6.1	0.069
*Weight (Kg)			71.3 ± 12.2	70.5 ± 13.5	0.782
*Height (Cm)			161.5 ± 7.5	162.8 ± 5.2	0.351
*BMI			27.2 ± 4.1	26.6 ± 4.9	0.472
*Gravid (N)			3.3 ± 1.6	3.2 ± 1.5	0.001
**Type of delivery (N/%)					
	C/S		2 (4.4%)	6 (13.3%)	0.313
	NVD		34 (75.6%)	32 (71.1%)	
**Education (N/%)					
	Primary		24 (53.3%)	14 (31.2%)	0.022
	Middle school		18 (40%)	20 (44.4%)	
	High educate		3 (6.7%)	11 (24.4%)	
**Job (N/%)					
		Housewife	38 (84.4%)	27 (60%)	0.034
		Clerck	4 (8.9%)	6 (13.3%)	
		Worker	3 (6.7%)	12 (27.6%)	

Data are presented as mean±SD.

* Student's t-test

** Chi-squared test.

**Table III:** Severity of variable in two groups (before and after intervention)

** Groups**			**Severe disorder**	**Sometimes Good**	**Most of good**	**Without disorder**	**p-value**
**Variable changes**							
Orgasm*							
	Physiotherapy group						0.001
		Before intervention	7 (15%)	18 (40%)	15 (34%)	5 (11%)	
		After intervention	1 (2%)	11 (24%)	21 (47%)	12 (27%)	
	Surgery group						0.001
		Before intervention	2 (5%)	11 (24%)	12 (27%)	20 (44%)	
		After intervention	9 (15%)	0	12 (25%)	27 (60%)	
Dyparunia*							
	Physiotherapy group						0.001
		Before intervention	13 (29%)	19 (42%)	8 (18%)	5 (11%)	
		After intervention	6 (13%)	12 (27%)	14 (31%)	13 (29%)	
	Surgery group						0.001
		Before intervention	32 (71%)	9 (20%)	4 (9%)	0	
		After intervention	37 (82%)	3 (7%)	5 (11%)	0	
Libido**							
	Physiotherapy group						0.007
		Before intervention	25 (56%)	13 (29%)	7 (15%)	0	
		After intervention	2 (4%)	20 (45%)	23 (51%)	0	
	Surgery group						0.001
		Before intervention	20 (44%)	13 (29%)	12 (27%)	0	
		After intervention	11 (25%)	14 (31%)	19 (42%)	1 (2%)	
Arousal**							
	Physiotherapy group						0.001
		Before intervention	4 (9%)	12 (27%)	16 (35%)	13 (29%)	
		After intervention	0	3 (6%)	21 (47%)	21 (47%)	
	Surgery group						0.001
		Before intervention	0	10 (22%)	24 (54%)	11 (24%)	
		After intervention	1 (2%)	10 (22%)	17 (38%)	17 (38%)	

Number are presented as N (%).

* Wilcoxon Rank test

** Fisher exact test.

## Discussion

The impact of UI and POP on a woman's life extends well but, the impact on sexual function has received little attention. However, up to 68% of women attending urogynecology clinics admit to experiencing sexual dysfunction and large prospective studies evidence have demonstrated that prolapse and/or incontinence adversely affects on sexual function (14-17). Sexual function improvement would result in improved women wellbeing, quality of life, and self-esteem. In a study in United States 19 to 59 percent of healthy women had sexual dysfunction and were sexually dissatisfied 68-75% (11). 

The levator ani muscle contracture results in elevation of intrapelvic organs and has a prompt role in protection and function of pelvic organs sphincters. The ischiocavernous muscle is attached to the clitoral cap and results in clitoris congestion and the bulbocavernous muscle compresses the dorsal deep vein of clitoris that would be persistent. The bulbocavernous and puborectalis muscles would increase the contact during intercourse with vaginal restriction. Contracture of Levator ani, bulbococcygeous, and ileococcygeous muscles would result vaginal congestion (12). 

In present study the libido improvement in both surgery and physiotherapy groups was significantly differed after intervention compared to before. Also the surgery resulted in a significant improvement of arousal, but had no effect on orgasm and increased dyspareunia was reported. In the study by Kuhn* et al,* the surgery of pelvic organs resulted in a significant improvement in libido, arousal, and dyspareunia but the orgasm was not affected (12). Pauls *et al* reported 98 patients under surgery for POP with no improvement in different stages of sexual dysfunction (18). 

In a review article by Achtari* et al* the effect of posterior vaginal repair on dyspareunia was evaluated in several studies that showed decreased dyspareunia after surgical repair of perineal body and increased dyspareunia after bulking the levator ani muscle (4). Azar *et al* study's in Iranian women showed that 67 women with POP were recruited in the study and FSFI questionnaire was used to assess sexual function of the cases preoperatively and 12-16 weeks after the operation. Results demonstrated that sexual function was improved postoperatively (19). 

In study by Komesu *et al* 73 patients with POP and underwent different surgical methods for prolapse and incontinence (vaginal abdominal), 30 women with posterior repair, and 43 without it. They concluded that repair of POP and UI would result in improved sexual function and posterior repair would result in increased dyspareunia without positive effect on sexual function (20). Hence regarding the similar findings between this study and other studies it may be said that in surgical methods the body image would be improved after anatomical correction that would result in increased libido (18, 12). 

The study by Kizilkaya *et al* demonstrated that physiotherapy would result in improvement of libido, orgasm, and dyspareunia but had no effect on arousal (21). Wurn *et al* performed physiotherapy in 29 patients and evaluated orgasm and dyspareunia in them and finally concluded that physiotherapy resulted in improvement of orgasm and dyspareunia (22). In a controlled trial Liebergall-Wischnitzer *et al* showed to assess the effectiveness of two exercise methods on SF and QoL in women suffering from SUI. The intervention included two exercise regimens: Paula method-12 weeks for 45 minutes sessions; pelvic floor muscle training (PFMT) 12 weeks of group (up to 10 participants) sessions of 30 minutes in length once a week, for 4 weeks plus two additional sessions, 3 weeks apart.

This study demonstrated that the Paula method of exercise was presented for the first time in the literature as a conservative noninvasive treatment for SUI and SF (23). In Present study, the physiotherapy resulted in a significant improvement in sexual arousal, orgasm, and dyspareunia compared to surgical method. Several studies have reported that enforcement of pelvic floor muscles would effectively treat the UI. Pelvic floor exercises would result in improved sexual function, that causes increasing vaginal blood flow and subsequent genital sensation, and it would result in improved arousal, orgasm, and libido (7, 22). 

The fact that powerful contractions of pelvic floor muscles may cluster urethra, therefore, increases intra urethral pressure, and prevents the urine leakage during an increase pressure in the intra-abdominal. In the cases of urge UI may inhibit reflexively or voluntarily the involuntary detrusore contraction (). Based on the few data available there is strong evidence for the efficacy of physical therapy for the treatment for SUI in women but further studies are needed to evaluate the optimal training protocol and length of treatment (13). 

Some studies have shown an improvement using a validated measure or no change or deterioration that used non-validated questionnaires in sexual function postoperatively. Rogers *et al* used a validated measure, while others used non-validated questionnaires (17, 25-28). Furthermore, other factors such as age, parity, cultural differences, previous pelvic surgery, and variations in surgical techniques may have an impact on sexual function. 

## Conclusion

In conclusion, the present study demonstrates that women reported a significant improvement in variables of sexual function, 2 months after surgery and physiotherapy for incontinence and prolapse as reflected by a significant change from the preoperative score. However, the degree of distress cannot be established because a condition-specific questionnaire measuring distress is not available at present but in this study, the FSFI was selected because it was used frequently in other studies, and it has Iranian validity and reliability (29, 30). Totally in this study, it seems that pelvic floor physiotherapy is an optimal method for sexual or psychological function improvement and may be a good alternative for surgery.
